# HYPERTENSIVE URGENCY IN OLDER ADULT WITHOUT HYPERTENSION HISTORY: THE DANGERS OF OVERTREATMENT WITH LEVOTHYROXINE

**DOI:** 10.1093/geroni/igad104.2966

**Published:** 2023-12-21

**Authors:** Vanessa Joyce Evardone, Jesssica Gendernalik, Ricardo Blondet, Sandra DiScala, Michael Silverman

**Affiliations:** Florida Atlantic University - West Palm Beach VA Medical Center, West Palm Beach, Florida, United States; Florida Atlantic University - West Palm Beach VA Medical Center, West Palm Beach, Florida, United States; West Palm Beach VA Medical Center, West Palm Beach, Florida, United States; West Palm Beach VA Medical Center, West Palm Beach, Florida, United States; West Palm VA Medical Center, West Palm Beach, Florida, United States

## Abstract

A 72-yr old woman with well-controlled depression and active lifestyle was seen at urgent care for “nervousness.” She was found to have borderline tachycardia and blood pressure of 187/114 mmHg. There were no other associated symptoms. Physical examination and labs were unremarkable, with normal renal function. EKG showed sinus tachycardia at 110 bpm. Recheck of BP after 30 minutes was 135/80mmHg, and she was discharged home on Lisinopril. Two days later, she again had “nervousness” and was taken to the Emergency Department. Blood pressure was 200/105 mmHg. Again, she denied any other symptomatology, and had unremarkable physical exam and labs. Repeat BP was improved, and she was discharged home with instruction to follow up with PCP. Medication list included Bupropion for depression which she has been taking and tolerating well for years, and Levothyroxine 25 mcg daily, which was started by her PCP 8 weeks prior, for TSH of 5.43 mIU/L and FT4 of 1.1 ng/dL. Thyroid hormone therapy was stopped during recent ED visit, and she has had no recurrence of her anxiety symptoms. Her BP has been checked daily at home and has been at baseline level.

